# Bimodal Radiotherapy with Active Raster-Scanning Carbon Ion Radiotherapy and Intensity-Modulated Radiotherapy in High-Risk Nasopharyngeal Carcinoma Results in Excellent Local Control

**DOI:** 10.3390/cancers11030379

**Published:** 2019-03-17

**Authors:** Sati Akbaba, Thomas Held, Kristin Lang, Tobias Forster, Philippe Federspil, Klaus Herfarth, Matthias Häfner, Peter Plinkert, Stefan Rieken, Jürgen Debus, Sebastian Adeberg

**Affiliations:** 1 Department of Radiation Oncology, Heidelberg University Hospital, Im Neuenheimer Feld 400, 69120 Heidelberg, Germany; 2 Heidelberg Institute for Radiation Oncology (HIRO), National Center for Radiation Research in Oncology (NCRO), Im Neuenheimer Feld 400, 69120 Heidelberg, Germany; 3 Heidelberg Ion-Beam Therapy Center (HIT), Department of Radiation Oncology, Im Neuenheimer Feld 450, 69120 Heidelberg, Germany; 4 National Center for Tumor diseases (NCT), Im Neuenheimer Feld 400, 69120 Heidelberg, Germany; 5 Department of Otorhinolaryngology, Head and Neck Surgery, University Hospital Heidelberg, Im Neuenheimer Feld 400, 69120 Heidelberg, Germany; 6 Clinical Cooperation Unit Radiation Oncology, German Cancer Research Center (DKFZ), 69120 Heidelberg, Germany

**Keywords:** nasopharyngeal cancer, carbon ion radiotherapy, bimodal radiotherapy, carbon ions, local control, recurrence patterns, survival, toxicity

## Abstract

*Background:* In this analysis, we aimed to present the first results of carbon ion radiotherapy (CIRT), which is known for its conformal dose distribution and increased biological effectiveness in the treatment of high-risk nasopharyngeal carcinoma (NPC). *Methods:* We retrospectively analyzed twenty-six consecutive patients who had been treated at our center with CIRT for high-risk NPC between 2009 and 2018. Carbon ion (C12) boost was applied in a bimodal setting combined with intensity-modulated radiotherapy (IMRT) base plan. The median cumulative total dose was 74 Gy (RBE), and patients with inoperable (*n* = 17, 65%) or incompletely resected (*n* = 7, 27%) tumors were included in the analysis. Overall, 81% received concomitant chemotherapy (*n* = 21). *Results:* The median follow-up time was 40 months (range 10–97 months) for all patients. At the last follow-up, 92% of the patients were still alive. We could identify excellent tumor response with complete tumor remission (CR) in 60% (*n* = 15/25), partial tumor remission (PR) in 20% (*n* = 5/25), and stable disease (SD) in 12% (*n* = 3/25) of the patients according to the RECIST (Response Evaluation Criteria in Solid Tumors) criteria. Despite unfavorable tumor characteristics, only one patient showed a locally in-field recurrence after 56 months (4%) and another patient a locoregional recurrence in the unilateral cervical lymph nodes after 21 months (4%). The 2-year local control (LC), distant progression-free survival (DPFS), and overall survival (OS) were 95%, 93%, and 100% and the estimated 5-year LC, DPFS, and OS were 90%, 86%, and 86%, respectively. Overall, treatment was tolerated well with 20% acute and 16% chronic grade 3 side effects. No toxicity greater than grade 3 occurred. *Conclusion:* Bimodal radiotherapy including IMRT and active raster-scanning CIRT for high-risk nasopharyngeal cancer is a safe treatment method resulting in moderate toxicity and excellent local control. A larger patient number and longer follow-up time would be necessary to strengthen the current findings.

## 1. Background

Nasopharyngeal malignancies amount to less than 1% of all tumors worldwide with an incidence of 84,000 and mortality of 51,000 annually; in addition, the incidence and mortality rates depend on sex, race, and geography [1]. Histology varies from squamous cell carcinoma (SCC) as the most common subtype to rare histologies, i.e., basaloid carcinoma, adenocarcinoma, adenoid cystic carcinoma (ACC), sarcoma, or mucosal melanoma. The World Health Organization (WHO) classifies nasopharyngeal carcinoma (NPC) into three subgroups: WHO type I for keratinizing tumors, WHO type II for non-keratinizing, differentiated tumors, and WHO type III for non-keratinizing, undifferentiated tumors including lymphoepithelial histology [2].

NPC is diagnosed mostly at an inoperable stage due to the late occurrence of symptoms, making radiation the therapy standard in the local treatment of nasopharyngeal malignancies [3,4]. Prognosis strongly depends on the tumor, node, metastasis (TNM)stage of the International Union Against Cancer (UICC) and the American Joint Committee on Cancer (AJCC), classifying nasopharyngeal tumors into five categories (UICC stage I–IVB) [3]. Thus, locoregional failure-free survival, distant failure-free survival, and overall survival (OS) decrease with increasing UICC stage [3]. For UICC stage I tumors, radiotherapy (RT) alone is seen as the first-line treatment [5,6]. For the upper UICC stages II–IVB, the addition of concomitant chemotherapy to the radiation reduces the occurrence of distant metastases, improves local control (LC), and results in significantly higher OS rates [7,8], thereby recommending chemoradiotherapy (CRT) for advanced stages [9,10,11]. While the use of induction and adjuvant chemotherapy is frequently discussed in the current literature, its role in LC and survival outcome is still unclear [7,11,12,13,14,15]. Due to the high propensity of NPC for early spread into bilateral cervical lymph nodes, all patients, despite negative lymph nodes, should be treated with bilateral neck irradiation [16,17].

With the development of more conformal RT techniques, like IMRT, a notable improvement in OS and toxicity could be achieved compared to two- and three-dimensional conformal RT [18,19,20,21]. Nevertheless, the proximity of these tumors to critical structures leads to considerable treatment-related side effects [8,22,23,24,25,26,27,28]. Thus, more conformal RT techniques are required to minimize the toxicity. Dosimetric comparisons between IMRT and proton beam radiotherapy have shown promising results, but clinical data for particle therapy are missing to date [29,30]. In the current study, we aimed to analyze the impact of CIRT—which is known for its conformal dose distribution and increased biological effectiveness—in combination with IMRT on local control and toxicity in the treatment of high-risk NPC as a further available RT technique.

## 2. Methods

### 2.1. Evaluation

Twenty-six patients with NPC who were treated with CIRT in a bimodal setting at the Heidelberg Ion-Beam Therapy Center (HIT, Heidelberg, Baden-Württemberg, Germany) for carbon ions (C12) and at the Department of Radiation Oncology, University Hospital Heidelberg for IMRT between 2009 and 2018 were analyzed retrospectively and OS, LC, local progression-free survival (LPFS), distant progression-free survival (DPFS), recurrence patterns of local relapse, and toxicity were assessed. Patients were selected for bimodal treatment in the case of insufficient dose distribution on the target or insufficient preservation of the organs at risk with IMRT. The study was conducted in accordance with the Declaration of Helsinki, and the protocol was approved by the Ethics Committee of the University Hospital Heidelberg (S-421/2015).

Patients were examined every three months during the first two years after completion of RT, every six months during the next two years, and then once a year with contrast-enhanced magnetic resonance imaging (MRI). Computed tomography (CT) of the chest was performed annually. Treatment response was evaluated by an ENT specialist according to the current Response Evaluation Criteria in Solid Tumors (RECIST) based on the MRI scans performed and the clinical examinations [31]. TNM and UICC stage was adjusted to the eighth edition of the TNM staging system of the UICC and the AJCC [3].

Toxicity was carried out according to the Common Terminology Criteria for Adverse Events (CTCAE) version 5. Acute toxicity was defined as treatment-related side effects that occurred during and within 3 months after RT and chronic side effects as treatment-related toxicity identified ≥3 months post-RT.

Time-to-event data (OS, LPFS, DPFS) were calculated from the first date of histopathological diagnosis to the last follow-up, death, or time of event (local or distant failure) by using the Kaplan–Meier method (IBM SPSS Statistics version 24). LC was assessed from RT up to the last follow-up or local failure. We refrained from univariate and multivariate analyses in order to determine prognostic factors for survival data because of the low patient number (*n* = 25/26, one patient was lost to follow-up) and the low incidence of events (*n* = 2 deaths, *n* = 1 local recurrence, *n* = 1 locoregional recurrence, *n* = 2 distant progression).

### 2.2. Patient Characteristics

Overall, twenty-six patients (58% females) with a median age of 49 years (range 28–73 years) who received bimodal RT for high-risk NPC could be identified. Twenty-five patients were available for survival analysis due to missing data for one patient (loss to follow-up). Eighty-one percent of the patients (*n* = 21) were treated for macroscopic tumor disease due to surgical inoperability (*n* = 17, 65%) or R2 resection (*n* = 4, 15%). The majority of the patients had tumors in advanced stages (T4, *n* = 16, 62%; T3, *n* = 7, 27%). Skull base infiltration could be identified in 38% of the patients (*n* = 10). Patient characteristics are described in Table 1.

### 2.3. Treatment Features

**Figure 1:**
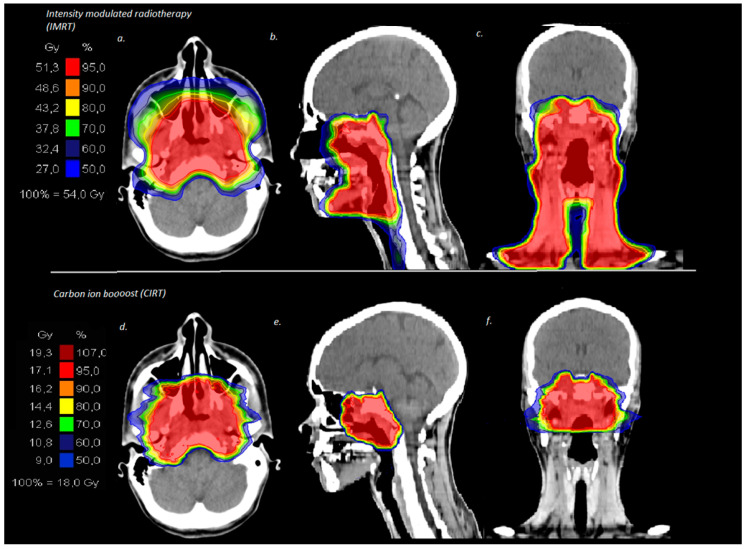
Bimodal treatment plan for a patient with T4 N2 nasopharyngeal cancer treated in a primary setting with intensity-modulated radiotherapy locally including the bilateral cervical lymphatic drainage (**a**–**c**) and carbon ion boost on the macroscopic tumor in the nasopharynx (**d**–**f**).

Treatment planning was performed with native as well as contrast-enhanced CT and MRI scans and patients were immobilized with individually performed thermoplastic head masks with shoulder fixation. Clinical target volume 1(CTV1) including the macroscopic tumor or prior tumor bed in the case of surgical resection and planning target volume 1 (PTV1) with a safety margin of 3 mm around the CTV1 were outlined for the C12 boost. Doses were prescribed on the CTV1 receiving ≥95% of the prescribed isodose. CTV2 included CTV1 and the bilateral cervical nodal levels I–III in the case of N0 and I–IV in the case of lymphonodal spread and, thus, corresponded at the same time to the PTV2. IMRT was conducted via Tomotherapy^®^ and IMRT doses were prescribed on CTV2 covering the CTV2 with at least 90% of the prescription dose. A bimodal treatment plan for a patient with a T4 N2 tumor is depicted in Figure 1. Cumulative median total dose was 74 Gy (RBE, relative biological effectiveness) (range 72–74 Gy (RBE)) corresponding to an equivalent dose in 2 Gy fractions (EQD2) of 80 Gy (range 78.5–80 Gy) and treatment volumes were large with a median CTV1 of 118 cc (range 24–324 cc) and a median CTV2 of 481 cc (range 131–1070 cc). Overall, 81% of the patients received concomitant chemotherapy (*n* = 21) during the time period of IMRT. The applied chemotherapy regimen and treatment characteristics are depicted in Table 1.

**Figure 2:**
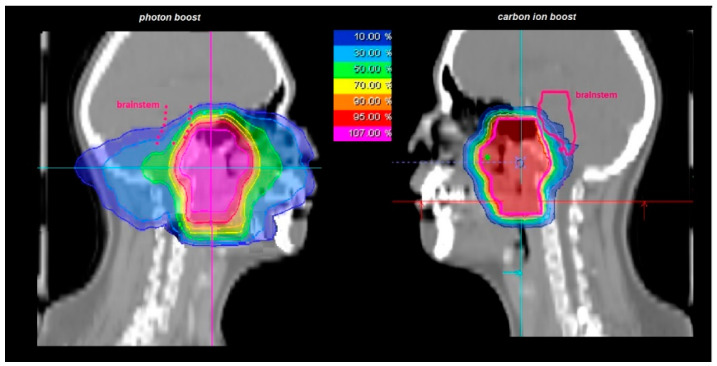
Photon boost plan and carbon ion boost plan for a patient with T4 staged nasopharynx carcinoma for comparison, especially regarding structures at risk. With carbon ions, more conformal doses can be delivered to the target organ and adjacent organs can be spared more adequately. While the brainstem receives at least 10% of the delivered dose in the photon boost plan, the brainstem can be spared nearly completely with carbon ion.

Dose constraints were defined with a tolerance dose for the spinal cord and eyes of maximum 45 Gy (EQD2) in summation, for the optic system and the brain stem a maximum of 54 Gy (EQD2), and for the unilateral parotid gland a mean of 26 Gy (EQD2) due to the proximity of NPC to critical structures following the QUANTEC data [32]. Carbon ions are known for a more conformal dose distribution of the target and more effective sparing of adjacent structures compared to photon beam RT. In Figure 2, you can see an irradiation plan for photon boost vs. carbon ion boost for a T4 staged nasopharynx carcinoma patient. While the brainstem is covered at least by the 10% prescription isodose in the photon boost plan, the brainstem can be spared nearly completely by carbon ions in the carbon ion boost plan.

## 3. Results

### 3.1. Survival Analysis

The median follow-up was 40 months (range 10–97 months) for all patients. At the last follow-up, 92% of the patients were still alive (*n* = 23/25, one patient was lost to follow-up). Overall, 60% of the patients showed a CR (*n* = 15/25), 20% a PR (*n* = 5/25), and 12% a SD (*n* = 3/25).

**Figure 3:**
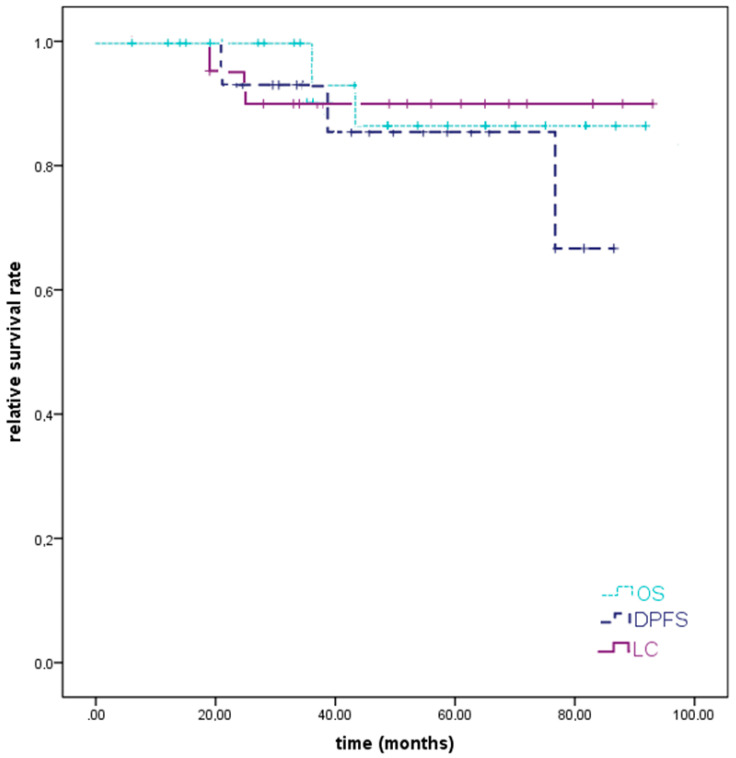
Kaplan–Meier estimates for local control (LC), distant progression-free survival (DPFS), and overall survival (OS). A 2-year LC, DPFS, and OS of 95%, 93%, and 100% and an estimated 5-year LC, DPFS, and OS of 90%, 86%, and 86% could be assessed for high-risk nasopharyngeal cancers after bimodal radiotherapy (RT).

In two patients, local or locoregional recurrent disease could be diagnosed (8%); in one patient, unilateral cervical lymph node metastases could be identified 26 months after the completion of primary RT for an initially T3 N0 G3 staged WHO I NPC, which was treated with salvage cervical neck dissection. At the last follow-up, the patient was still alive without any new diagnosed local or locoregional progressive disease. In another patient who was treated for a T4 N2 G3 WHO II NPC with skull base infiltration, an in-field recurrence was identified 51 months after RT. Pulmonary metastases occurred simultaneously. Thus, the patient received re-RT locally and systemic treatment for lung metastases and died 8 months later. Distant progression could be identified in 13% of the patients (*n* = 3) and involved the lung solely in one case (4%), the lung and the liver simultaneously in one case (4%), and the mediastinal lymphatic drainage in another case (4%). We could identify a 2-year LC, DPFS, and OS of 95%, 93%, and 100% and an estimated 5-year LC, DPFS, and OS of 90%, 86%, and 86%, respectively (Figure 3).

### 3.2. Toxicity

RT was tolerated well without side effects greater than grade 3. Overall, we could identify grade 3 acute toxicity in 20% (*n* = 5) and grade 3 chronic toxicity in 16% (*n* = 4) of the patients. The most reported grade 3 acute side effects were mucositis (*n* = 5, 20%), dysphagia (*n* = 4, 16%), and odynophagia (*n* = 4, 16%) with the need for supportive nutrition via gastric tube for acute side effects in 24% of the patients (*n* = 6). Three months after RT, the majority of these symptoms had resolved and no patient was further dependent on a gastric tube. An overview of acute and chronic toxicity is shown in Table 2. One patient with acute keratoconjunctivitis sicca developed vision impairment grade 3 six weeks after radiotherapy (4%). Overall, acute and late cranial nerve palsy accounted for 8% (*n* = 2) and 16% (*n* = 4) of the patients consisting of CRT-induced hearing impairment during therapy without the need of a hearing device during follow-up (8%), a mild effect on the abducent nerve (cranial nerve (CN) IV) 3 months and >24 months post RT (*n* = 2, 8%), a moderate effect on the hypoglossal nerve (CN XII) 24 months post RT (*n* = 1, 4%), and a moderate effect on the facial nerve (CN VII) >24 months after RT (*n* = 1, 4%). The median time to occurrence of cranial nerve palsy was 25 months (range 0–38 months). A hearing device was obligatory in one patient for severe hearing impairment due to tympanic effusion 12 months after RT (4%). Concerning brain injury, a blood-brain barrier damage grade 1 with T2/flair hyperintense changes of the temporal lobe in the follow-up MRI was observed 14 months after RT. In another patient, a symptomatic blood-brain barrier disorder of the temporal lobe with reported headache and dizziness 35 months after radiotherapy was treated with cortisone.

## 4. Discussion

CIRT was applied in patients with unfavorable tumor characteristics. All patients had high-risk carcinomas of the nasopharynx, which consisted of 69% of tumors in upper UICC stage III (*n* = 18) and involved the skull base in 38% (*n* = 10). Nevertheless, an excellent 5-year LC, DPFS, and OS of 90%, 86%, and 86% could be achieved. Despite large treatment volumes, CIRT was tolerated with moderate toxicity. No grade >3 toxicity could be identified. According to the grade 3 toxicity, 20% of the patients showed acute and 16% chronic grade 3 side effects.

Combined modality treatment with RT up to a total dose of 70 Gy to 72 Gy (EQD2) to the primary tumor and concomitant chemotherapy with cisplatin either 100 mg/m^2^ on days 1, 22, and 43 or 30–40 mg/m^2^ weekly for WHO I–III tumors represents the current standard of care for NPC [7,9,10,11,33,34]. RT is mostly applied with IMRT due to its dose conformity resulting in superior survival rates and less toxicity compared to 2D- and 3D-RT techniques [18,19,20,21]. Although dosimetric comparisons between IMRT and alternative RT techniques like proton beam RT have shown promising results, clinical data regarding proton beam radiotherapy and CIRT of NPC in a primary setting are lacking [29,30]. Although little data concerning carbon ion RT as salvage treatment for recurrent NPC are available [35,36], the current study will be, to our best knowledge and despite its retrospective character and limiting patient number, the first study reporting the clinical experience of CIRT applied in NPC in a primary setting with higher doses than described in the literature (median total dose in the current study was 72 Gy (RBE) corresponding to a total EQD2 of 80 Gy). Additionally, patients received guideline-appropriate treatment with concomitant cisplatin 40 mg/m^2^ weekly during IMRT.

In the current literature, 5-year OS rates between 71% and 84%, depending on the T, N, and UICC stage, as well as on the EBV-DNA levels, are described for advanced WHO nasopharyngeal tumors treated with RT or CRT [37,38,39,40]. Thus, we could identify excellent OS for this patient population with bimodal therapy including IMRT and CIRT in the current analysis (5-year OS of 86%). Additionally, meta-analyses and randomized studies described 5-year failure-free survival rates between 72% and 75% and 10-year PFS rates between 39% and 52% for patients with advanced WHO tumors treated with the same or similar combined treatment regime applied in the current study (RT up to 72 Gy, concomitant chemotherapy with cisplatin) [7,12,13,41,42,43]. In contrast, we observed excellent and much higher local and distant control rates, as mentioned. In only one patient with a skull base infiltrating tumor could we identify a local recurrent disease in-field. Even as salvage RT, carbon ions seem to be effective. Hu et al. reported excellent local control and survival rates with a 1-year OS and local recurrence-free survival of 98% and 87% for NPC patients who received re-RT with carbon ions for recurrence [35].

Besides ACC histology, which is known for its radio resistance and high local and locoregional spread among all tumor histologies in the head and neck area, skull base infiltration seems to be another risk factor for local failure with decreased local control rates in head and neck tumors [44,45,46,47]. In a large retrospective study including 7251 patients, Huang et al. established risk competing risk monograms for NPC patients predicting a higher cause-specific mortality for patients with WHO I/II NPC (vs. WHO III), advanced T or N stage, high EBV-DNA level, high lactate dehydrogenase (LDH) level, and high C-reactive protein (CRP) level [48].

Mucositis is seen as the most common acute toxicity after CRT of NPC. Al-Sarraf et al. could identify in a randomized phase-III study an acute grade 3 and 4 mucositis rate of 37% for patients treated with IMRT and concomitant cisplatin 100 mg/m^2^ on days 1, 22, and 43 [8]. Chen et al. described in another randomized trial an acute mucositis rate of 40% after CRT with concomitant cisplatin 40 mg/m^2^ weekly chemotherapy. The therapy was tolerated with low toxicity. Due to its dose conformity, we observed considerably decreased acute toxicity with grade 3 acute mucositis in 20% of our patients (*n* = 5) and a rate of gastric tube dependence for nutrition difficulties attributable to mucositis, dysphagia, odynophagia, or appetite loss of 24% after bimodal RT (*n* = 6). The majority of our patients received concomitant chemotherapy (81%, *n* = 21/26) and, thus, we identified a higher rate of acute and late toxicity compared to prior experiences regarding skull base infiltrating tumors that were treated with heavy ions without concomitant chemotherapy in our center [49]. Nevertheless, mucositis (85% grade 1–3 mucositis) and xerostomia (48% grade 1–3 xerostomia) represented the most common and xerostomia the most long-lasting acute toxicity in the current analysis. Overall, long-term xerostomia was observed in eight patients (32%), showing a lower xerostomia rate with carbon ions in comparison to conventional and intensity-modulated RT. In the current literature, late xerostomia of 41% to 62% with 0% to 3% ≥grade 3 xerostomia and of 90% to 97% with up to 24% ≥grade 3 xerostomia are described for IMRT and conventional RT [50,51,52,53,54].

Several severe late side effects after RT of tumors close to the skull base can occur up to 20 years after treatment [24]. Temporal lobe necrosis, osteoradionecrosis, and cranial nerve palsy can be considered as the most serious chronic side effects of RT for these tumors. Temporal lobe necrosis occurs predominantly 1–3 years after treatment in nearly 3% of the patients and increases with higher RT doses and the volume affected by high RT doses [27,55,56,57,58,59]. Schlampp et al. described 5% and 50% probability of developing temporal lobe necrosis for heavy ions when RT doses of 68.8 Gy (RBE) and 87.3 Gy (RBE) were applied on the skull base [58]. A similar dose dependence could be observed for photons as well. Thus, Huang et al. identified a 5-year temporal lobe necrosis rate of 13.2% after IMRT, which increased for higher prescribed doses [60]. Although higher doses were applied in the current study, rates of brain barrier damage (8%) were lower compared to photon data (6%–18%) [50,53].

Regarding cranial nerve palsy, Geara et al. described cranial nerve palsy to occur up to 21% 5 years after photon beam RT and Kong et al. identified cranial nerve palsies in 45% of their patients within 20 years after photon RT [61,62]. In addition, Chew et al. reported about bulbar palsy with moderate and severe functional loss after photon beam RT up to 18 years after RT in nearly 20% of the patients [24]. Several authors described a dose dependence of cranial nerve palsy after particle therapy [63,64]. Demizu et al. identified significantly more optic nerve palsy when doses of 65 Gy (RBE) were applied to the optic nerve [64]. Urie et al. described 1% cranial nerve complications at 60 Gy (RBE) and 5% at 70 Gy (RBE) [63]. During a median follow-up of 38 months, we could observe an acute and late effect on the cranial nerves ≤ grade 2 in overall 2 (8%) and 4 patients (16%) with a median latency of 25 months. Cranial nerve palsy can occur many years after RT. Considering the actual database, this rate can increase with longer follow-up. However, Urie et al. reported an occurrence of more than 50% of the diagnosed cranial nerve palsies within the first 24 months and none after 60 months after RT [63]. Therefore, a longer follow-up will be necessary to make a clear statement in this matter.

Despite a negative patient selection, high RT doses and large RT volumes applied in the current analysis, bimodal RT with IMRT and CIRT results in excellent local control rates and moderate toxicity.

## 5. Conclusions

First results of chemoradiotherapy with combined dose-escalated active raster-scanning carbon ion radiotherapy and intensity-modulated radiotherapy for high-risk nasopharyngeal cancer show excellent local control and survival outcome with favorable treatment-related morbidity compared with historical controls. Nevertheless, a larger number of patients and a prospective study design are necessary to evaluate further a dose escalation.

## Figures and Tables

**Table 1:** Patient, disease, and treatment characteristics, *n* = 26.

Characteristic	No. (%)
**median age**	49 (28–73) years
**gender**	
male	11 (42)
female	15 (58)
**Karnofsky performance score in %**	
100	6 (23)
90	9 (35)
80	5 (19)
70	4 (15)
60	2 (8)
**nicotine abuse**	
yes	9 (35)
no	17 (65)
**alcohol abuse**	
yes	5 (19)
no	21 (81)
**histology**	
WHO I	3 (12)
WHO II	7 (27)
WHO III	16 (62)
**UICC stage**	
I	1 (4)
II	1 (4)
III	6 (23)
IVA	15 (58)
IVB	3 (12)
IVC	none
**TNM stage**	
T1	2 (8)
T2	1 (4)
T3	7 (27)
T4	16 (62)
T4 with skull base infiltration	11 (42)
N0	8 (31)
N+	14 (54)
Nx	4 (15)
M0	26 (100)
**definitive RT**	17 (65)
**postoperative RT**	9 (35)
Rx	2 (8)
R1	3 (12)
R2	4 (15)
**therapy regimes**	
50 Gy/2 Gy IMRT + 24 Gy/3 Gy (RBE) C12	5 (19)
56 Gy/2 Gy IMRT + 18 Gy/3 Gy (RBE) C12	21 (81)
**median total dose**	74 Gy (RBE) (72–74 Gy (RBE))
**median CTV1**	118 cc (24–324 cc)
**median CTV2**	481 cc (131–1070 cc)
**concomitant chemotherapy**	0
cisplatin 40 mg/m^2^ weekly	19 (73)
cisplatin and fluorouracil	1 (4)
carboplatin and fluorouracil	1 (4)

Abbreviations: CTV = clinical target volume, C12 = carbon ions, IMRT = intensity-modulated radiotherapy, RBE = relative biological effectiveness, RT = radiotherapy, TNM = tumor, nodal, metastasis stage, UICC = Union Internationale Contre le Cancer, WHO = World Health Organization.

**Table 2:** Overview of acute and chronic toxicity, *n* = 25.

Characteristic	Acute Toxicity, No. (%)	Chronic Toxicity, No. (%)			
	Under RT and Until 6 Weeks Post RT	3–6 Months Post RT	12 Months Post RT	24 Months Post RT	At Last Follow-Up
**toxicity**					
<grade 3	21 (84)	14 (56)	11 (44)	8 (32)	9 (36)
=grade 3	5 (20)	3 (12)	4 (16)	4 (16)	4 (16)
>grade 3	0	0	0	0	0
**mucositis**					
grade 1	6 (24)	4 (16)	0	0	0
grade 2	12 (48)	0	0	0	0
grade 3	5 (20)	0	0	0	0
**dermatitis**					
grade 1	8 (32)	3 (12)	0	0	0
grade 2	5 (20)	0	0	0	0
grade 3	1 (4)	0	0	0	0
**dysphagia**					
grade 1	5 (20)	4 (16)	3 (12)	3 (12)	3 (12)
grade 2	7 (28)	1 (4)	0	0	0
grade 3	4 (16)	0	0	0	0
**odynophagia**					
grade 1	5 (20)	4 (16)	0	0	0
grade 2	8 (32)	0	0	0	0
grade 3	4 (16)	0	0	0	0
**hyposmia**					
grade 1	3 (12)	3 (12)	3 (12)	0	0
grade 2	3 (12)	1 (4)	0	0	0
grade 3	1 (4)	0	0	0	0
**xerostomia**					
grade 1	8 (32)	9 (36)	8 (32)	7 (28)	7 (28)
grade 2	7 (28)	4 (16)	1 (4)	1 (4)	1 (4)
**gastric tube dependence**	6 (24)	1 (4)	0	0	0
**keratoconjunctivitis sicca**					
grade 1	1 (4)	1 (4)	1 (4)	1 (4)	1 (4)
grade 2	3 (12)	1 (4)	0	0	0
grade 3	1 (4)	1 (4)	1 (4)	1 (4)	1 (4)
**tympanic effusion**					
grade 1	9 (36)	7 (28)	7 (28)	5 (20)	5 (20)
grade 2	8 (32)	3 (12)	3 (12)	2 (8))	2 (8)
**cranial nerves affected**					
auditory nerve					
grade 2	2 (8)	0	0	0	0
abducent nerve					
grade 1	0	1 (4)	0	0	1 (4)
hypoglossal nerve					
grade 2	0	0	0	1 (4)	0
facial nerve					
grade 2	0	0	0	0	1 (4)
**temporal lobe necrosis**					
grade 1	0	0	1 (4)	0	0
grade 2	0	0	0	0	1 (4)

Abbreviations: CRT = chemoradiotherapy, RT = radiotherapy.

## Data Availability

All data generated or analyzed during the current study are included in this published article. The dataset is available from the corresponding author on reasonable request.

## Abbreviations

ACC	adenoid cystic carcinoma
AJCC	American Joint Committee on Cancer
CIRT	carbon ion radiotherapy
CR	complete remission
CRT	chemoradiotherapy
CT	computed tomography
CTCAE	Common Terminology Criteria for Adverse Events
C12	carbon ions
DPFS	distant progression-free survival
EQD2	equivalent dose in 2 Gy fractions
HIT	Heidelberg Ion-Beam Therapy Center
LPFS	local progression-free survival
LC	local control
IMRT	intensity-modulated radiotherapy
MRI	magnetic resonance imaging
NPC	nasopharyngeal carcinoma
OS	overall survival
PR	partial remission
RECIST	Response Evaluation Criteria in Solid Tumors
RT	radiotherapy
SD	stable disease
TNM	tumor, node, metastasis
UICC	International Union Against Cancer
WHO	World Health Organization
